# Exploring Spectrogram-Based Audio Classification for Parkinson’s Disease: A Study on Speech Classification and Qualitative Reliability Verification

**DOI:** 10.3390/s24144625

**Published:** 2024-07-17

**Authors:** Seung-Min Jeong, Seunghyun Kim, Eui Chul Lee, Han Joon Kim

**Affiliations:** 1 Department of AI & Informatics, Graduate School, Sangmyung University, Hongjimun 2-gil 20, Jongno-gu, Seoul 03016, Republic of Korea; 202333025@sangmyung.kr (S.-M.J.); 202134015@sangmyung.kr (S.K.); 2 Department of Human-Centered Artificial Intelligence, Sangmyung University, Hongjimun 2-gil 20, Jongno-gu, Seoul 03016, Republic of Korea; 3 Department of Neurology, Seoul National University College of Medicine, Seoul National University Hospital, Daehak-ro 101, Jongno-gu, Seoul 03080, Republic of Korea

**Keywords:** PSLA, AST, explainable AI, Parkinson’s disease, speech classification

## Abstract

Patients suffering from Parkinson’s disease suffer from voice impairment. In this study, we introduce models to classify normal and Parkinson’s patients using their speech. We used an AST (audio spectrogram transformer), a transformer-based speech classification model that has recently outperformed CNN-based models in many fields, and a CNN-based PSLA (pretraining, sampling, labeling, and aggregation), a high-performance model in the existing speech classification field, for the study. This study compares and analyzes the models from both quantitative and qualitative perspectives. First, qualitatively, PSLA outperformed AST by more than 4% in accuracy, and the AUC was also higher, with 94.16% for AST and 97.43% for PSLA. Furthermore, we qualitatively evaluated the ability of the models to capture the acoustic features of Parkinson’s through various CAM (class activation map)-based XAI (eXplainable AI) models such as GradCAM and EigenCAM. Based on PSLA, we found that the model focuses well on the muffled frequency band of Parkinson’s speech, and the heatmap analysis of false positives and false negatives shows that the speech features are also visually represented when the model actually makes incorrect predictions. The contribution of this paper is that we not only found a suitable model for diagnosing Parkinson’s through speech using two different types of models but also validated the predictions of the model in practice.

## 1. Introduction

Parkinson’s disease (PD) is the second most common neurodegenerative disorder worldwide, primarily resulting from the progressive loss of neurons in the substantia nigra of the midbrain, leading to a deficiency of dopamine produced by these neurons. This deficiency results in impaired motor functions and progressive non-motor symptoms such as cognitive impairments [1]. Epidemiologically, the prevalence and incidence of Parkinson’s disease increase with age. According to a study by Willis et al. [2], the incidence rates among North Americans are 108 to 212 per 100,000 for those aged 65 and above and 47 to 77 per 100,000 for those over 45. Similarly, research by Park et al. [3] noted significant disparities in the South Korean population, where the incidence rate for individuals under 49 is 1.7 per 100,000, with a prevalence of 5.8, whereas those aged 50 and above have an incidence rate of 88.7 per 100,000 and a prevalence of 455.7. Parkinson’s disease poses substantial public health and economic burdens, particularly in countries experiencing population aging. In the United States, the annual cost attributed to PD is estimated at approximately USD 52 billion [2]. In South Korea, the total medical insurance costs related to PD reached KRW 542.8 billion in 2020, marking a 25.3% increase from four years earlier [4]. This financial burden extends not only to the nations but also to patients suffering from the disease and their families who bear the caregiving responsibilities.

One of the prominent symptoms among PD patients is speech and articulation disorders. Many experience hypokinetic dysarthria, characterized by reduced movement, resulting in rapid speech, diminished vocal loudness, monotone rhythm, and altered speech patterns [5]. Cognitive impairments can further deteriorate patients’ speech capabilities, manifested through reduced sentence and vocabulary comprehension, attention deficits, and overall impaired language function [5]. These issues could serve as biomarkers for screening the disease. Various studies have identified acoustic features such as tVSA, VAI, noise-to-harmonics ratio (NHR), jitter, and pitch from vowel phonation and demonstrated their diagnostic relevance [6,7,8]. Similarly, features like the average articulation rate and syllable duration stability, short-term variability measured by Jitt and nPVI, rate slope, and relative amplitude of vowels and consonants in diadochokinetic (DDK) tasks have been effectively used as biomarkers [9,10,11,12]. Recent advances include applying machine learning models to these acoustic features or using deep learning for automated classification of PD, showing notable performance [13,14,15].

However, in the field of AI learning for medical applications, including PD classification, there are issues related to data bias, which primarily stem from difficulties encountered during data collection [16,17,18]. Specifically, when collecting voice data from PD patients, the challenges faced by those with cognitive impairments in performing multi-tasks, the essential requirement for patient consent, and the fragmented healthcare systems restrict researchers to a limited sample of patients. These challenges not only limit the amount of data collected but also hinder the collection of high-quality data, thereby increasing the bias and reducing the performance of deep learning models. Moreover, deep learning possesses inherent “black box” characteristics. Like in other industries, transparency and trust in decision-making are crucial in healthcare, as the performance of models and the understanding of how conclusions are reached can significantly impact patient health and treatment outcomes. Furthermore, the lack of interpretability is closely related to ethical considerations, which are of paramount importance to regulatory bodies like the FDA [19]. Therefore, there has been a significant push in the medical field to develop interpretable forms of machine-learning models [20].

In this study, we aimed to identify distinguishing patterns between Parkinson’s patients and healthy controls using two prominent models in general voice classification: the transformer-based AST [21] and the PSLA [22] model, which incorporates CNNs and attention modules. Quantitative comparisons show that PSLA outperforms AST, with 92.15% accuracy and 97.43% AUC, compared to 87.75% accuracy and 94.16% AUC by AST. The study also explores the application of explainable AI (XAI) techniques such as class activation mapping (CAM) [23], Gradient-weighted class activation mapping (Grad-CAM) [24], and Eigen-CAM to address the challenges of the black-box problem by providing interpretability to deep learning predictions. Our qualitative analysis using the Eigen-CAM [25] algorithm offers insights into the reasons behind the model’s predictions, further validating its reliability and effectiveness in distinguishing between the groups based on vocal characteristics, specifically capturing the narrow speech bandwidth and distinct articulation features of PD patients.

## 2. Related Works

### 2.1. Deep Learning-Based Speech Classification Research

Recent studies have presented new approaches to detect and classify speech changes in Parkinson’s disease (PD) patients using deep learning. These studies explore the potential of speech signal analysis in diagnosing PD, with a particular focus on key symptoms of PD patients, such as hypokinetic slurred speech. Wodzinski et al. [26] presented a novel method for detecting PD through vowel analysis of continuous speech utterances utilizing the ResNet architecture. They converted the spectrum of voice recordings into a method used for image classification and used a pre-trained ResNet to classify Parkinson’s patients, with extensive time-domain augmentation to ensure dataset diversity and avoid overfitting. The study, using the PC-GITA database, consisted of 100 participants (50 healthy controls and 50 diagnosed with PD), with each participant recorded three times. The study achieved an accuracy of over 90% on the validation set, demonstrating that PD can be detected from the spectrogram of the speech signal.

A deep dual learning ensemble model was designed by Jie et al. [27]. In this model, a deep sample learning algorithm and a deep network (deep feature learning) were combined to realize deep dual learning of PD speech data. First, an embedded stack group sparse autoencoder was designed to perform deep feature learning and obtain new high-level deep feature data; second, through the L1 normalized feature selection method, the deep features were fused with the original speech features to form hybrid feature data. Third, an iterative mean clustering algorithm (IMC) was designed to construct a deep sample learning algorithm and perform deep sample transformation. Then, a hierarchical sample space was constructed based on the deep sample learning algorithm, and a classification model was built on top of this space. Finally, a weight fusion mechanism was designed to integrate the classification model into an ensemble model, which fused the deep feature learning algorithm and the deep sample learning algorithm together. This ensemble model was named the deep dual learning ensemble model. At the end of the study, it was validated using two representative speech datasets for PD. The experimental results show that the average accuracy of the proposed algorithm is 98.4% and 99.6%, respectively, which is better than the existing state-of-the-art algorithms. This study demonstrates the superiority of deep dual learning over traditional deep feature learning for PD speech recognition.

Quan et al. [28] explored the feasibility of speech change detection for PD diagnosis by including both static and dynamic speech characteristics. In particular, they noted significant differences in articulatory transition characteristics, such as transition counts and fundamental frequency trends, between healthy controls and Parkinson’s patients. Based on these findings, they proposed a method to capture the time-series dynamic characteristics of speech signals using a bidirectional long short-term memory (LSTM) model. The method was evaluated using 10-fold cross-validation and dataset partitioning with no overlapping individual samples and significantly improved the accuracy of PD detection over traditional methods using only static features (DT acc 73.46% → bidirectional LSTM acc 84.29%).

In another study, Quan et al. [29] proposed a novel end-to-end deep learning model for detecting PD from speech signals to address the problem of hypokinetic unclear pronunciation, which is common in more than 90% of PD patients. The model used a time-distributed two-dimensional convolutional neural network (2D-CNN) to extract dynamic features from time-series data and applied a one-dimensional CNN (1D-CNN) to analyze the dependencies between these features. Validated on two different databases, the model outperformed expert trait-based machine learning models, achieving 81.6% and 75.3% accuracy on the sustained vowel /a/ and short Chinese sentence reading tasks in Database-1, respectively, and up to 92% accuracy on speech tasks reading simple (/loslibros/) and complex (/viste/) sentences in Spanish in an evaluation of different sound types including vowels, words, and sentences in Database-2. By visualizing the features generated by the model, the learned time-series dynamic features captured the overall frequency range and reduced variability that are key features of Parkinson’s sounds, providing important clinical evidence for the detection of Parkinson’s patients. In particular, the paper argues that the low-frequency region of the mel-spectrogram is more important and influential than the high-frequency region for Parkinson’s detection in speech.

Giovanni et al. [30] compared various machine learning (ML) techniques, integrating traditional ML algorithms with a convolutional neural network (CNN) architecture custom-designed for deep learning. Algorithms such as KNN, SVM, and naïve Bayes were evaluated, with KNN performing slightly better. The study also highlighted the effectiveness of correlation-based feature selection (CFS) in identifying the most relevant vocal biomarkers. These biomarkers proved to be instrumental in differentiating between healthy individuals, untreated early-stage Parkinson’s patients, and patients with more advanced stages treated with L-Dopa. Overall, studies have shown that both feature-based ML approaches and deep learning techniques yield similar results in classifying different stages of PD.

Asmae et al. [31] introduced the SMOTE technique to address the problem of class imbalance. They performed attribute selection using the chi-square attribute selection technique to select the most important attributes. They selected three deep learning classifiers: long short-term memory (LSTM), bidirectional LSTM (Bi-LSTM), and deep LSTM (D-LSTM). After tweaking the parameters by selecting different options, our experiments showed that the D-LSTM outperformed the LSTM and Bi-LSTM techniques. They achieved high accuracy scores of 94.87% and 97.44% on the unbalanced and balanced original datasets, respectively.

Existing speech research on PD classification uses traditional methods to train and test classification models rather than fully state-of-the-art models such as LSTMs, one-dimensional CNNs, and ResNets. However, for general speech classification models, there are studies that use structures to achieve higher performance, such as transformers that use attention to focus on specific parts of speech rather than simple deep learning models. Therefore, we conducted an investigation to select a model that can learn speech features better than the existing simple type of deep learning models. The summary of previous works is summarized in Table 1.

### 2.2. EXplainable AI (XAI)

XAI is becoming increasingly important as the number of complex artificial intelligence models grows in various industries, especially in sensitive areas such as healthcare and finance. The main focus of XAI is to make the model’s decision-making process transparent, which helps build trust in the model’s predictions. In healthcare, the need for explainability is critical for regulatory approval of various AI models for patient safety. A representative method of XAI is CAM, which was described in a 2016 paper [23], which applied global average pooling (GAP) instead of a fully connected layer to generate a heatmap of a specific class image and claimed that the heatmap could be used to understand how the CNN predicted the image to be a specific class. In the GAP layer, global average pooling is performed for each feature map, resulting in a vector of the same length as the number of channels in the feature map entering the GAP layer. The GAP is followed by the FC layer, which takes the vector output from the GAP as input to obtain the final heat map. Despite its very simple structure, the feature map that passes through the last convolution layer is quite accurate because it contains all the contents of the input image. However, this has the disadvantage that CAM cannot extract heat maps from feature maps in the middle rather than the last feature map, and it must use a global average pooling layer followed by a fully connected layer. Grad-CAM can be used without the GAP layer as a way to overcome the shortcomings of CAM. The weight of each feature map, which is weighted in CAM, is transferred using a gradient instead. Then, like CAM, it performs a pixel-wise sum and applies the ReLU function to select the parts that find positive weights to generate a heatmap. The later Eigen-CAM can generate heatmaps without having to change the structure of the network or compute noisy gradients like CAM. It also proposed a method to localize objects well, regardless of adversarial examples. Compared to conventional CAM and Grad-CAM, Eigen-CAM has the advantage of localizing multiple objects and localizing objects in the background. Finally, it is robust to adversarial examples. Therefore, in this study, we used Grad-CAM and Eigen-CAM, which are more robust to various types of models, to XAI the imaged speech data.

## 3. Methods

### 3.1. Data Preprocessing

The dataset employed in this study includes speech recordings from Korean individuals and is structured to distinguish between healthy controls and patients diagnosed with PD, following the Movement Disorders Society clinical diagnostic guidelines [34]. The dataset comprises equal numbers of participants, 100 diagnosed with PD and 100 healthy individuals, engaged in various speech tasks that require sustained and repetitive speech patterns.

Consent was duly obtained from all participants, and the study protocol was rigorously reviewed and approved by the Institutional Ethical Review Board (IRB No.: 2108-235-1250). The control group demographics included 47 males and 53 females, with an average age of 65.8, while the PD group consisted of 49 males and 51 females, with an average age of 64.3 years. Participants with PD had an average disease duration of 6.9 years and an average Hoehn and Yahr stage of 1.9, indicating mild to moderate disease severity. Comprehensive demographic and clinical details are outlined in Table 2.

This research involved three specific speech simulation tasks relevant to PD classification: the production of vowels, the production of consonants, and the DDK task. Each task was designed to capture the unique vocal patterns of the participants, which include both Parkinson’s patients and healthy controls.

The vowel task required participants to repeat the sequence of vowels ‘/a/, /e/, /i/, /o/, and /u/’ two times. In the consonant task, participants articulated a series of Korean phonemes such as /ga-ga-ga/, /na-na-na/, /da-da-da/, and /ha-ha-ha/ three times consecutively. The DDK task, commonly known as the ‘pa-ta-ka’ sequence, involved rapid pronunciation of the sequence for 10 s following a deep breath. Summary information related to voice tasks can be found in Table 3 below.

Recordings were meticulously gathered using the “Voice Recorder Pro 3.7.1” app on an iPhone, within a carefully controlled environment to minimize background noise. All audio files were recorded at a frequency of 16,000 Hz and at a bitrate of 64 kbps. This methodical approach ensured the integrity and clarity of the speech data crucial for subsequent analysis. The duration of tasks varied, with the control group averaging 88.37 ± 20.79 s, and the Parkinson’s group slightly longer at 93.30 ± 19.30 s, reflecting subtle differences in speech dynamics between the groups.

The preprocessed speech is converted into log Mel filterbank features. These features, used as input images for the PSLA and AST models, are derived by compressing the frequency axis of the Mel-spectrogram into a log scale and then quantizing it into multiple stages of bins. The energy in each bin is calculated by aggregating the energy across the corresponding frequency bands. This feature map allows for the observation of energy changes in both the time domain and frequency domain, thus providing higher dimensional representation than one-dimensional raw audio data or two-dimensional waveform data. Such a representation facilitates more effective learning processes in machine learning [35].

**Figure 1:**
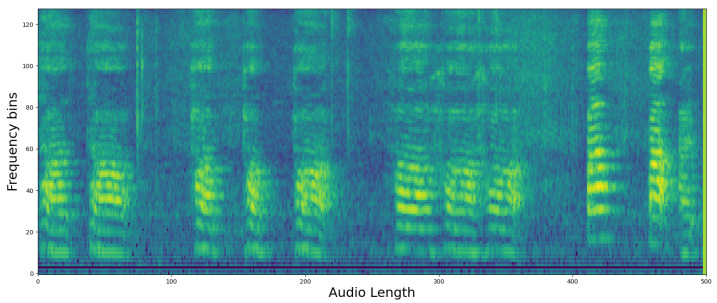
Example of a Mel filterbank visualization.

Furthermore, by compressively representing the frequency axis compared to the traditional Mel-spectrogram, the log Mel filterbank significantly enhances both spatial and temporal efficiency during neural network training. Specifically, the transformation of these features utilizes a 25 ms Hanning window with a 10ms overlap on the time axis (*x*-axis), and on the frequency axis (*y*-axis), the maximum frequency of 8000 Hz for a 16,000 Hz audio file is converted into the log Mel scale and then quantized into 128 bins. Below is an example of the extracted log Mel filterbank features, as shown in Figure 1.

### 3.2. Model Training

In this study, we aimed to classify PD patients and healthy controls using the high-performance audio classification models AST and PSLA. For AST, the transformed 128-d log Mel filterbank features are divided into 16 × 16 patches with an overlap of 6 units. Each divided patch is linearly embedded into a vector of length 768, and to allow the model to learn data location and class information, a trainable position embedding layer of the same length is added to the patch embedding, with a [CLS] token appended. Since AST is designed for classification tasks, we used only the encoder of the transformer. We employed the standard architecture available in PyTorch for the transformer encoder, which consists of an embedding dimension of 768, 12 layers, and 12 multi-head attention modules.

**Figure 2:**
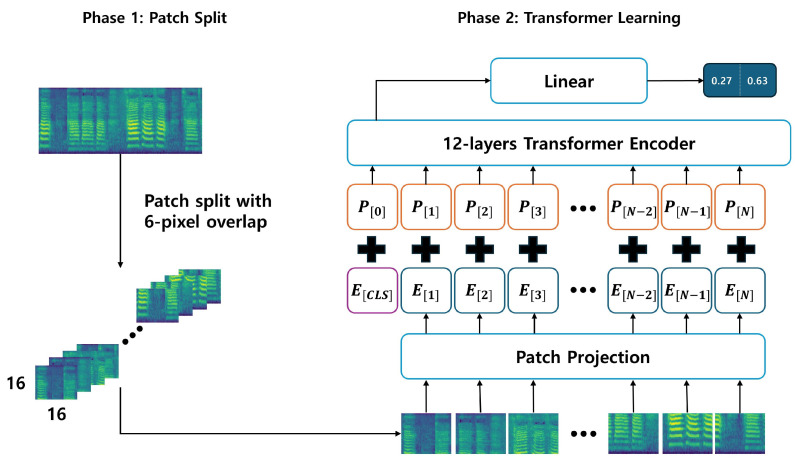
The structure of AST.

Unlike the 3-channel image input of ViT, the log mel filterbank features consist of single-channel spectrograms. We averaged the weights corresponding to each of the three input channels in the ViT patch embedding layer to adapt them for the AST patch embedding layer and normalized the input audio spectrogram so that the dataset’s mean and standard deviation were 0 and 0.5, respectively. Finally, we employed knowledge distillation to learn the CNN’s inductive biases using the weights of DeiT [36] pre-trained on ImageNet [37]. The outcomes from DeiT include a [CLS] token and a [DIST] token, and in this study, we projected the average of these two tokens through an MLP layer to predict the most likely class. The structure of AST is shown in Figure 2.

**Figure 3:**
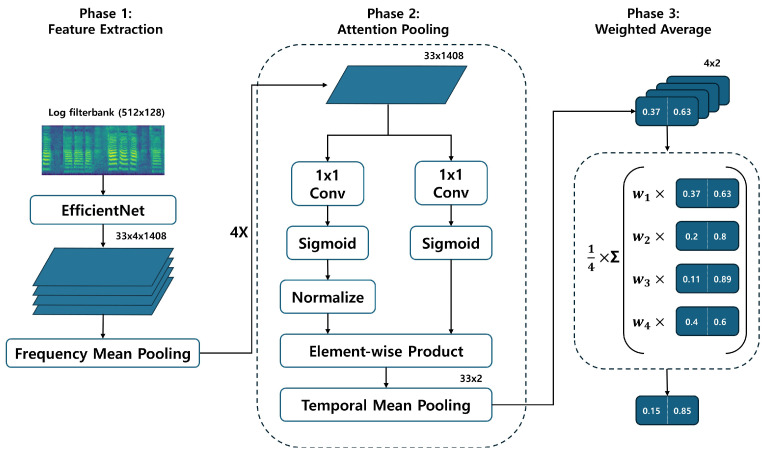
The structure of PSLA.

For PSLA, although it follows the same data normalization assumptions as AST, it does not undergo patch division but instead inputs the image directly into an EfficientNet [38] pre-trained on ImageNet. Subsequent features extracted are then averaged pooled along the frequency axis, and a 4-head attention module is used to facilitate the model’s understanding of the feature map and to predict probabilities for each class. Specifically, the attention module transforms the channel dimension of the feature map to match the class size through two parallel 1 × 1 kernel convolution layers. After normalization is applied to one of the resulting feature maps and multiplied by the other, the final prediction is computed by applying a weighted average through learned weights for each head after average pooling along the time axis. The structure of PSLA is shown in Figure 3.

During the model development phase, we programmed the algorithm to undergo 50 epochs for both the training and validation phases to ensure robust model training. The model that exhibited optimum performance during the validation phase was subsequently selected for further assessments on an independent test dataset. This systematic training strategy allowed for comprehensive model evaluation.

In the processing of each 5 s audio segment, a log mel filterbank was utilized with a configuration of 512 time samples and 128 frequency bins. The learning rate was set to 0.00001, and we adopted the Adam optimizer to facilitate better convergence during training. To further enhance the variability of the training data, we implemented data augmentation techniques such as frequency and time masking [39]. Additionally, Mixup [40] was applied at a ratio of 0.5 to augment the dataset diversity. Given the minor discrepancies in segment counts across classes, the balanced sampling feature of AST was considered but ultimately found unnecessary for this specific dataset.

For the PSLA model, training, validation, and testing were conducted on the same dataset splits as those used for AST. The primary encoder used in PSLA was the EfficientNet-B2, which was pretrained on ImageNet, and the model utilized four attention heads. The training involved 50 epochs to provide adequate learning time. The Adam optimizer was chosen to facilitate optimal convergence, with a learning rate set to 0.001.

Similar to AST, data augmentation techniques such as frequency and time masking were implemented to enhance the dataset’s complexity. Mixup was also applied with a probability of 0.5 to increase the diversity of the training examples. Also, like AST, balanced sampling was not employed in the PSLA model due to the minor variations in segment counts across classes.

### 3.3. XAI

**Figure 4:**
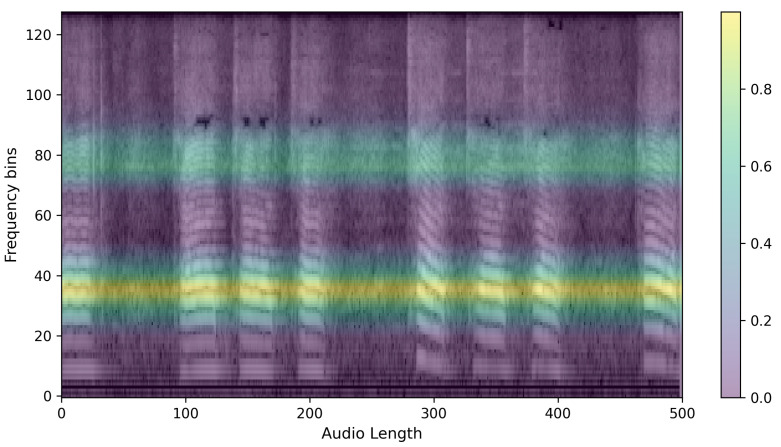
Example of heatmap representation of significant regions using EigenCAM technique on log Mel filterbank image.

Finally, to evaluate the reasonableness of the model’s predictions, we compared the predicted results for each model by outputting them as heatmaps. Since our training data is in the form of images, we used CAM techniques commonly used in image prediction models and compared the predicted heatmaps using Grad-CAM, Grad-CAM++, and EigenCAM, as mentioned in Section 2.2. Of the three XAI CAMs, we determined that EigenCAM had the highest explanatory power for the same output, so we ran our evaluation through EigenCAM. An example of a heatmap for a single piece of speech data is shown in Figure 4. The figure shows a single spectrogram imaged by EigenCAM from the PSLA prediction model. The spectrogram of the speech data is represented in grayscale, and the heatmap is represented using the color map on the right. The higher the value, the more focused the model is in making predictions, and the lower the value, the more unfocused the model is. As can be seen in Figure 4, for the PSLA model, the heatmap regions are formed along the frequency axis.

## 4. Experiments and Results

### 4.1. Quantitative Classification Model Evaluation

#### 4.1.1. Evaluation Metrics

We measured accuracy, sensitivity, specificity, f1 score, area under the curve (AUC), equal error rate (EER), and d-prime ($d^{\prime }$) to measure the performance of the speech-based PD classification model. Accuracy is a ratio that indicates how many samples out of all samples the model correctly classified (in Equation (1)) and is a basic indicator that allows you to intuitively understand the overall performance of the model. Sensitivity refers to the proportion of people who actually have PD that the model correctly predicts to have PD (in Equation (2)). High sensitivity means that it can identify most patients with the disease. In the case of specificity, it is the proportion of people who correctly predict that there is no disease among those who do not have the disease (in Equation (3)). High specificity can minimize cases where the model misdiagnoses healthy people. In addition, since both precision and recall are important in classification problems, the average performance of the two indicators was evaluated through the F1-score (in Equation (4)), which is the harmonic mean of the two indicators.

AUC represents the area under the receiver operating characteristic (ROC) curve and measures whether high performance can be maintained regardless of the threshold in the trade-off relationship between true positive rate and false positive rate. EER refers to the ratio of the point where the false positive rate (FPR) and false negative rate (FNR) become equal, and in the case of D-prime, it quantifies the possibility of distinguishing between the two states. D-prime ($d^{\prime }$) is the difference in the mean of the probability distribution between cases with PD and cases without PD expressed in standard deviation units (in Equation (5)). The higher this indicator is, the more effectively the classification model can distinguish between the two states. The formulas for the evaluation indicators are as follows:

(1) $$\mathrm{Accuracy}=\frac{TP+TN}{TP+TN+FP+FN}$$

(2) $$\mathrm{Sensitivity}=\frac{TP}{TP+FN}$$

(3) $$\mathrm{Specificity}=\frac{TN}{TN+FP}$$

(4) $$F1\text{-}\mathrm{Score}=2\times \frac{\mathrm{Precision}\times \mathrm{Recall}}{\mathrm{Precision}+\mathrm{Recall}}$$

(5) $$d^{\prime }=\sqrt{2}\;\Phi^{-1}\left(\mathrm{AUC}\right)$$

In the above formula, TP represents the number of true positive samples, TN is the number of true negative samples, FP is the number of false positive samples, and FN is the number of false negative samples. Additionally, $\Phi^{-1}$ refers to the inverse function value of the standard normal distribution value.

#### 4.1.2. Model Performance Comparison

**Figure 5:**
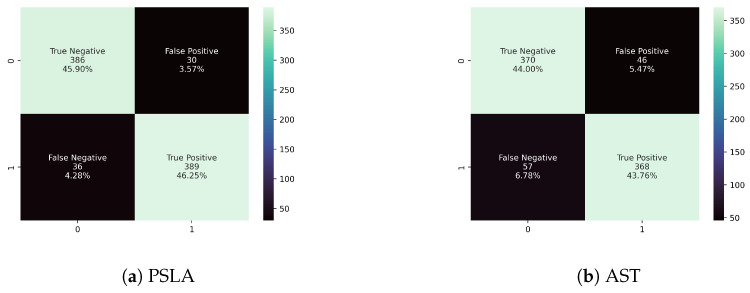
Confusion matrix of (**a**) PSLA and (**b**) AST. The second line of each block of the confusion matrix represents the number of test negative samples belonging to each case, and the third line represents the ratio based on the number of samples.

As shown in Table 4, a comparative analysis between the AST and PSLA models highlights significant disparities in key performance metrics. The PSLA model outperforms the AST, achieving an accuracy of 92.15% compared to AST’s 87.75%. In sensitivity, PSLA reaches 91.53%, surpassing AST’s 86.59%, and in specificity, it registers 92.79%, bettering the 88.94% of AST. Furthermore, the confusion matrix presented in Figure 5 illustrates PSLA’s superior performance across all classes compared to AST. These performance metrics are crucial for the accuracy of medical diagnoses, demonstrating PSLA’s enhanced reliability in clinical applications.

**Figure 6:**
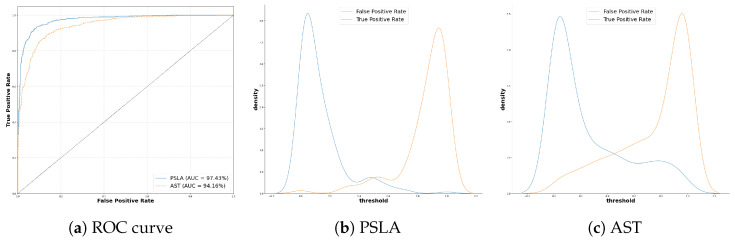
(**a**) ROC curve and KDE plot of (**b**) PSLA and (**c**) AST.

Furthermore, the PSLA model demonstrates superior discriminative ability with an AUC of 97.43%, compared to 94.16% for the AST model. Additionally, PSLA exhibits a lower EER of 7.73% and a higher d-prime value of 2.754, further underscoring its robustness. The ROC curve and kernel density estimation (KDE) graphs for the true positive rate (TPR) and false positive rate (FPR), depicted in Figure 6, illustrate these distinctions. The ROC curve for PSLA is notably positioned toward the upper left, indicating a more efficient threshold setting, while the KDE comparison reveals a more distinct inter-class distribution than AST. These metrics collectively affirm PSLA’s enhanced precision and reliability in clinical settings, especially in speech classification tasks, indicating its potential utility in medical diagnostics.

**Figure 7:**
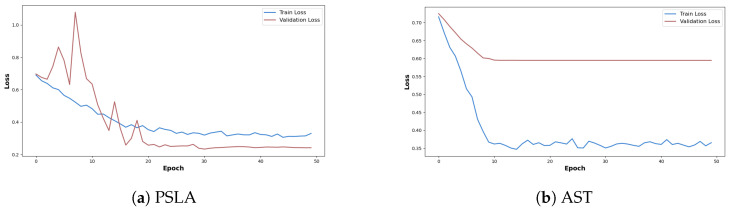
Loss curves for the training and validation of (**a**) PSLA and (**b**) AST.

**Figure 8:**
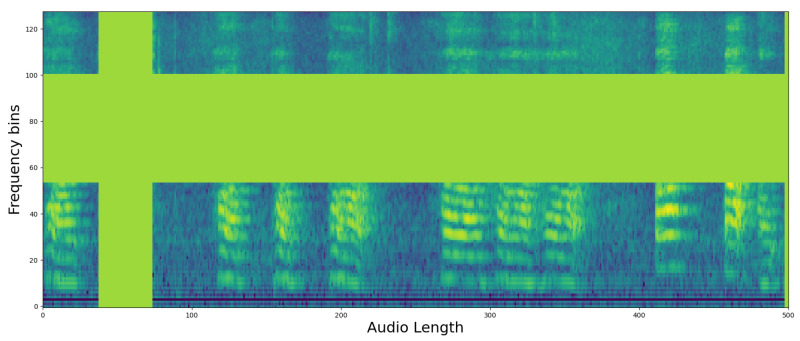
Example of a heatmap representation of significant regions using the EigenCAM technique on log Mel filterbank image with Specaugment.

In Figure 7, the loss curves during the learning and verification process of the two models are recorded. In the case of AST, the training loss continues to fall, while the validation loss plateaus from the 10th epoch, and there is no longer improvement, while in the case of PSLA, the variation in validation loss is relatively large, but it can be seen that it continues to decrease as the training loss decreases. Another point among the resulting graphs is that the training loss is lower than the validation loss in Figure 7a. This can be seen as a result of the augmentation technique we used in our study. We used the Specaugment [39] technique to augment the mel-spectrogram images we generated. We used this technique to increase the amount of data by masking certain time and frequency bands in the mel-spectrogram image to increase the amount and diversity of training data. An example of masked data using Specaugment is in Figure 8. However, in validation, we used the original unprocessed and clean mel-spectrogram image for prediction, which resulted in a higher loss in the training set than in the validation set. In addition, in AST, the difference between the convergence point of training loss and validation loss is quite large, whereas in the case of PSLA, not only is there little difference in the convergence point of validation loss and training loss, but the validation loss is actually lower. Through these results, it was confirmed that AST shows a tendency to overfit in small datasets, and on the contrary, PSLA proved to extract important features for classifying normal and PD groups even in small datasets.

Finally, we conducted experiments to evaluate the time complexity and memory complexity of two deep learning models. The results indicated that PSLA exhibited overall more efficient performance compared to AST. As shown in Table 5, with a batch size of 8, AST required over 1 min per epoch during the training process, whereas PSLA completed an epoch in 40 s. Additionally, during the inference stage, AST exhibited a latency of approximately 3.8 s, while PSLA demonstrated a latency of approximately 1.9 s, showing about a two-fold difference. In terms of GPU memory consumption, AST utilized approximately 2.8 times more memory than PSLA, indicating a significant dependence on GPU resources.

Upon synthesizing the quantitative results, it is evident that the PSLA model outperforms the transformer-based AST model in speech-based clinical experiments. This finding suggests that in environments characterized by limited datasets, the PSLA model excels as a feature extractor for distinguishing between two distinct classes, whereas the AST model exhibits comparatively lower effectiveness. Under similar constraints, the PSLA model emerges as the preferable choice for tasks that demand high precision and reliability, underscoring its potential as a robust tool in clinical settings.

### 4.2. Qualitative Classification Model Evaluation

**Figure 9:**
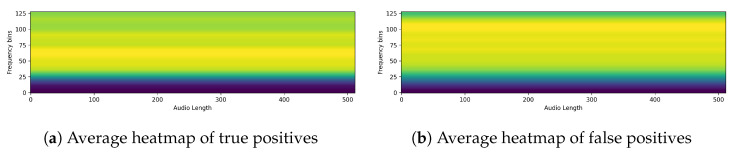
Average heatmap of (**a**) true positives and (**b**) false positives.(A lighter yellow color in the heatmap means the model is more focused, and a darker blue means it is less focused.)

**Figure 10:**
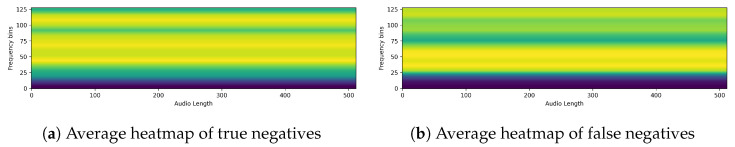
Average heatmap of (**a**) true negatives and (**b**) false negatives.(A lighter yellow color in the heatmap means the model is more focused, and a darker blue means it is less focused.)

Next, we ran XAI on PSLA, a model with good predictive performance, through EigenCAM. We then extracted the average of the heatmap for each section of the confusion matrix. The images below show the heatmaps for each prediction of true positive, false positive, true negative, and false negative averaged over time and frequency, respectively. From Figure 9 and Figure 10, we can clearly see that the model is separately focusing on frequencies above and below the median frequency that would have been determined to be normal. This suggests that for speech predicted to be Parkinson’s, the model focuses and discriminates more evenly over the frequency domain. The heatmap shown in the following figures represents a variety of color spectra, with the closer to yellow indicating higher density. The heatmap is calculated from the final layer of the model, and the horizontal lines allow you to see which frequencies the model indicates as the basis for determining the presence or absence of Parkinson’s disease. For example, the heatmap of a specific PD patient shows strong intensity in the high-frequency band, which suggests that the model is based on the main speech characteristics of PD patients that show poor quality in the high-frequency band [8].

**Figure 11:**
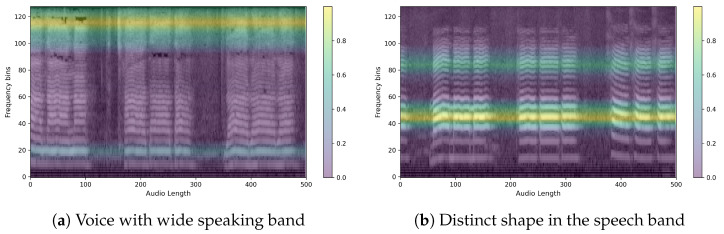
Sample heatmaps of the control group. (Mel-Spectrogram overlaid with a heatmap of what the model is focusing on.)

**Figure 12:**
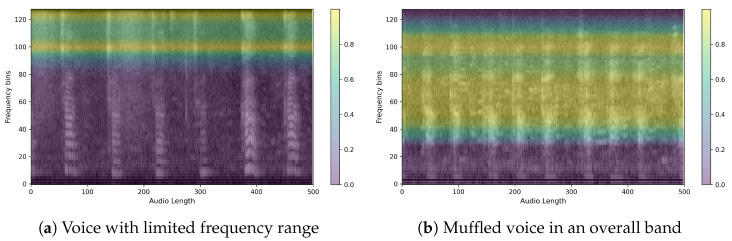
Sample heatmaps of the PD group. (Mel-Spectrogram overlaid with a heatmap of what the model is focusing on.)

Here are example images for each true positive, false positive, true negative, and false negative. First is the control speech, which is when the model’s prediction is actually correct. As can be seen in Figure 11, the speech has a wide speaking band and a distinct shape in the speaking band. We can also see that the speech is stable and has a clear power sequence with no breaks in the speech. The CAM also presented the firing frequency of the high and mid-band as the basis for the judgment, and it was possible to confirm that the waveform was evenly distributed in both bands. Figure 12 is Parkinson’s speech, and the actual model prediction is correct. In both examples, we can see that the waveform of the voice is uneven compared to that of a normal person. We can also see that the speech is characteristically more pronounced in the low bass band, but the overall band is muffled. In fact, the CAM showed that the model focused on high frequencies, which is where the uneven waveform of the voice is located.

**Figure 13:**
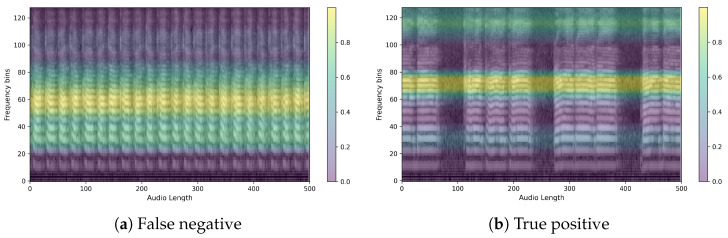
Sample for comparison of two predictions for a PD patient with the same ID.

Finally, we further analyzed CAM for cases where different predictions were made for the same person. First, we show an example of a person with Parkinson’s in Figure 13. The voice was predicted to be a normal person on the left, Figure 13a, and a Parkinson’s patient on the right, Figure 13b.

Notice the different focus areas of the models. In fact, the control group is characterized by sharpening of the voice shape, while the PD group is characterized by crushing of the voice shape. This can be clearly seen by looking at the model’s focus area for the same person’s voice. The image on the left, Figure 13a, shows the PD group but predicted as a control group, while the image on the right, Figure 13b, shows the PD group predicted correctly. In fact, when we check the model’s focus area through the heatmaps when the model predicts the PD group as a control group, it seems to focus on the mid-band from 20 to 90, where the voice pattern is clearly observable. On the other hand, when predicting the PD group, the model can be said to have identified the area where the voice is present through the 60–80 range, which is the normal range of the voice, and identified the distorted shape from the high-frequency band 100–120, which is the area where the voice is characterized by a muffled sound.

**Figure 14:**
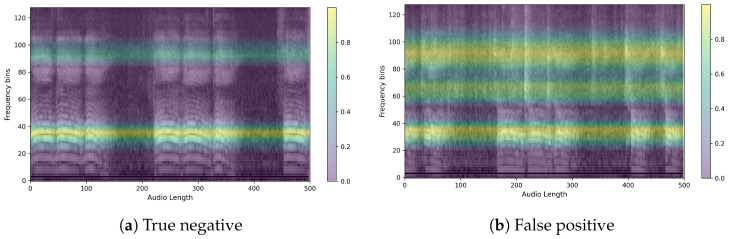
Sample for comparison of two predictions for a non-PD patient with the same ID.

Another example is for control subject 66. It is the same person but predicted differently. It can be seen that the one on the left, Figure 14a, has an evenly clean waveform across multiple bands, while the one on the right, Figure 14b, has a distorted waveform in the high frequencies. In fact, when we checked the frequencies that the model focused on using CAM, we found that it focused on the two frequency bands with cleaner waveforms for TN, while for FP, the heatmap was dominated by a clean low-band area and two places where the waveform was not clean. From these two examples, we see that the model looks for clean waveforms in both the low and high bands when predicting the control group. We can also infer that when predicting the dementia group, the model identifies the presence of speech and the pattern of speech by looking not only at the frequency bands where the waveforms are distorted but also at the bands where the waveforms appear clean. This gives us an idea of what the model is focusing on as it learns to image the features of speech and how it makes its judgments.

## 5. Discussion

We conducted additional experiments to validate the effectiveness of filtering out silent segments longer than 3 s in data preprocessing. As shown in Table 6, the AST model showed a significant improvement in accuracy of more than 8% after filtering compared to before filtering, and sensitivity, which is important in the medical field, showed an increase of more than 10%. This significant improvement indicates that removing non-informative segments can enhance the model’s ability to correctly identify speech features relevant to PD.

Similarly, PSLA in Table 7 also showed a small but significant performance improvement after filtering, highlighting the benefit of preprocessing steps in speech data analysis. This shows that in the case of speech data, non-significant bins interfere with the model’s learning and prediction. To further improve the model’s performance, we changed the model’s encoder from EfficientNet-b0 to EfficientNet-b2 to increase the complexity of the model. As a result, the specificity of the model increased by more than 3%, and the prediction performance for the normal group was strengthened. These improvements underscore the effectiveness of our proposed modifications in enhancing model accuracy and reliability. Through these experiments, we confirmed that preprocessing steps such as data filtering are necessary to extract relevant features for distinguishing between Parkinson’s and normal speech data. Additionally, we observed that training with refined data using a more complex model or a model pretrained on a general audio dataset could lead to further performance improvements.

## 6. Conclusions

In this study, we demonstrated promising performance in predicting Parkinson’s disease (PD) using patients’ voice data. The model achieved an accuracy of 92.15% and an AUC of 97.43%. These results suggest that the model has favorable properties for predicting PD when speech data are transformed into 2D log Mel filterbank images. Additionally, we employed eXplainable AI (XAI) techniques, such as GradCAM, GradCAM++, and EigenCAM, to visualize which parts of the voice spectrogram the model focuses on. Our findings indicate that CAM techniques performed meaningfully from a qualitative perspective, especially when analyzing the same individual.

The obtained heatmaps revealed distinct features between PD and non-PD patients. For control predictions, there are neat high and low-frequency bands, with EigenCAM focusing on both. Conversely, PD predictions are characterized by a muffled waveform, particularly in the high-frequency band, where EigenCAM focuses the most. This confirms that the trained model accurately reflects the characteristics of PD patients, such as having a choppy waveform across all bands.

Meanwhile, we recognize the importance of analyzing individual speech tasks independently to enhance model performance and insights. Although the current study segmented speech data into 5 s intervals to capture sufficient acoustic information, we did not separate tasks for individual analysis. Future work will involve detailed experiments to process and analyze each type of speech task separately, followed by early and late fusion techniques to combine the results. This approach is expected to provide deeper insights into the specific contributions of each task to the overall performance of PD classification.

Moreover, an ablation study on model and data preprocessing was conducted, but experiments on the parameters of the mel-spectrogram and the input image were not performed. This suggests that there is room for further performance improvement along with the model development, which will be addressed in future research.

Future work will also focus on developing predictive models for various diseases beyond PD and analyzing how XAI can elucidate the features of each disease. By extending our methodology and analysis to different conditions, we aim to enhance the generalizability and applicability of our models in the medical field.

## Figures and Tables

**Table 1:** Summary of related works.

Authors	Years	Dataset	Input Feature	Model	Accuracy
Wodzinski et al. [26]	2019	PC-GITA	Mel-spectrogram	CNN	91.7%
Jie et al. [27]	2021	LSVT voice rehabilitation dataset [32], Sakar dataset [33]	Original speech features/hybrid feature data	Embedded deep-stacked group sparse autoencoder (EGSAE)	98.4% and 99.6%
Quan et al. [28]	2021	Database collected from GYENNO SCIENCE	BBE, MFCC, DMFCC, DDMFCC	Bidirectional LSTM	84.29%
Quan et al. [29]	2022	Database collected from GYENNO SCIENCE, PC-GITA	Log Mel-spectrogram	2D-CNN + 1D-CNN	GYENNO: 81.56%, PC-GITA: 92%
Giovanni et al. [30]	2023	266 healthy controls (HCs) and 160 PD subjects (custom dataset)	Extracted features from various sources, Mel-spectrogram	Various machine learning models (KNN, SVM, naïve Bayes classifiers)/CNN	
Asmae et al. [31]	2023	Database collected by Max A. Little et al.	Acoustic features of the Parkinson’s dataset	Bidirectional LSTM	98.72%

**Table 2:** Characteristics of participants in the study, including demographics and clinical information.

	Control	PD
Age *	65.8 ± 7.6	64.3 ± 9.4
**Gender**		
Male	47	49
Female	53	51
**Disease duration (yr)**	Not applicable	6.9 ± 4.5
**HY stage**	Not applicable	1.9 ± 0.8
**Total Participants**	100	100

* Regarding gender characteristics, there is no statistically significant difference with *p* > 0.05.

**Table 3:** Speech tasks for Parkinson’s disease detection.

Task	Length	Remarks
/Pa/-/ta/-/ka/	~10 s	Repeat /pa/, /ta/, /ka/ alternately
Consonant	Various	Sequentially produce each consonant three times
Vowel	Various	Repeat /a/, /e/, /i/, /o/, /u/ alternately

**Table 4:** Quantitative performance comparison results of AST and PSLA.

Model	Accuracy	Sensitivity	Specificity	AUC	F1-Score	EER	d-Prime
AST	87.75%	86.59%	88.94%	94.16%	87.75%	12.84%	2.218
PSLA	92.15%	91.53%	92.79%	97.43%	92.15%	7.73%	2.754

**Table 5:** Time and memory efficiency comparison of models AST and PSLA.

Model	AST	PSLA
**Training Time**	60.14 s/epoch	38.92 s/epoch
**Inference Time**	3.8 s	1.9 s
**GPU Memory**	6.26 GB	2.24 GB

**Table 6:** Results of the ablation study on the AST model.

Method	Accuracy	Sensitivity	Specificity	AUC	F1-Score	EER	d-Prime
AST baseline	79.30%	76.11%	82.64%	87.30%	79.30%	20.93%	1.613
Filtered data +Audioset Pretraining	87.75%	86.59%	88.94%	94.16%	87.75%	12.84%	2.218

**Table 7:** Results of the ablation study on the PSLA model.

Method	Accuracy	Sensitivity	Specificity	AUC	F1-Score	EER	d-Prime
PSLA baseline	90.38%	91.15%	89.58%	96.66%	90.38%	9.50%	2.592
+Data filtering	90.61%	91.52%	89.66%	95.90%	90.60%	9.51%	2.459
+EfficientNet b2	92.15%	91.53%	92.79%	97.43%	92.15%	7.73%	2.754

## Data Availability

The raw data supporting the conclusions of this article will be made available by the authors on request.
